# Use of over-the-counter malaria medicines in children and adults in three districts in Kenya: implications for private medicine retailer interventions

**DOI:** 10.1186/1475-2875-6-57

**Published:** 2007-05-10

**Authors:** Timothy O Abuya, Wilfred Mutemi, Baya Karisa, Sam A Ochola, Greg Fegan, Vicki Marsh

**Affiliations:** 1 Kenya Medical Research Institute/Wellcome Trust Centre for Geographic Medicine Research -Coast, 80108, P.O. Box 230, Kilifi, Kenya; 2 Division of Malaria Control, Ministry of Health, 00100 GPO, P.O Box 20750, Nairobi, Kenya; 3 Ministry of Health, Kilifi District Hospital, 80108, P.O Box 9, Kilifi, Kenya; 4 Infectious Diseases Epidemiology Unit, Department of Epidemiology and Population Health, London School of Hygiene and Tropical Medicine, Keppel Street, London, WC1E 7HT, UK; 5 Centre for Tropical Medicine, University of Oxford, John Radcliffe Hospital, Headington, Oxford, OX3 9DU, UK

## Abstract

**Background:**

Global malaria control strategies highlight the need to increase early uptake of effective antimalarials for childhood fevers in endemic settings, based on a presumptive diagnosis of malaria in this age group. Many control programmes identify private medicine sellers as important targets to promote effective early treatment, based on reported widespread inadequate childhood fever treatment practices involving the retail sector. Data on adult use of over-the-counter (OTC) medicines is limited. This study aimed to assess childhood and adult patterns of OTC medicine use to inform national medicine retailer programmes in Kenya and other similar settings.

**Methods:**

Large-scale cluster randomized surveys of treatment seeking practices and malaria parasite prevalence were conducted for recent fevers in children under five years and recent acute illnesses in adults in three districts in Kenya with differing malaria endemicity.

**Results:**

A total of 12, 445 households were visited and data collected on recent illnesses in 11, 505 children and 19, 914 adults. OTC medicines were the most popular first response to fever in children with fever (47.0%; 95% CI 45.5, 48.5) and adults with acute illnesses (56.8%; 95% CI 55.2, 58.3). 36.9% (95% CI 34.7, 39.2) adults and 22.7% (95% CI 20.9, 24.6) children using OTC medicines purchased antimalarials, with similar proportions in low and high endemicity districts. 1.9% (95% CI 0.8, 4.2) adults and 12.1% (95% CI 16.3,34.2) children used multidose antimalarials appropriately. Although the majority of children and adults sought no further treatment, self-referral to a health facility within 72 hours of illness onset was the commonest pattern amongst those seeking further help.

**Conclusion:**

In these surveys, OTC medicines were popular first treatments for fever in children or acute illnesses in adults. The proportions using OTC antimalarials were similar in areas of high and low malaria endemicity. In all districts, adults were more likely to self-treat with OTC antimalarial medicines than febrile children were to receive them, and less likely to use them in recommended ways. Government health centres were the most common second resort for treatment and were often used within 72 hours. In view of these practices, more research is needed to assess the impact on the popularity of private medicine sellers of strengthened public sector policies on access to malaria treatment and insecticide-treated bed nets. Improved targeting of OTC antimalarials to high risk groups, better communication strategies regarding adult as well as children's dosages, and facilitating more rapid referral to trained health workers where needed are important challenges to private medicine seller programmes.

## Background

Malaria continues to be a major cause of death and disability [1] and contributes to economic weaknesses in poor countries [2] despite increased international support for, and coordination of, research and control efforts [3,4]. Insecticide-treated bed nets (ITNs) and intermittent presumptive treatment of malaria in pregnant women and infants have been shown to be successful control measures, and research on malaria vaccines holds promise for the future [4-7]. However, the availability of prompt effective treatment of clinical malaria will remain a fundamental control mechanism either alone or alongside additional measures, where these can be effectively implemented [4]. There are macroeconomic and political challenges in ensuring that effective antimalarial medicines are available through the public sector at country level [8-10]. In Kenya, for example, implementation of antimalarial drug policy changes in 2005 occurred more than 32 months after the initial decision, as a result of complex financing and procurement systems and prolonged negotiations with the pharmaceutical industry [11]. Socio-economic and geographic barriers to treatment through government clinics and hospitals has led to a proliferation of untrained private medicine sellers in many settings [12]. In recognition of the latter phenomenon and the problems associated with practices in this sector, international and national malaria control strategies have identified private medicine sellers as an important target to promote appropriate home treatment [13,14].

Since 2001, the Kenya National Malaria Strategy (KNMS) has supported the implementation of programmes targeting private sellers of OTC medicines and their clients within district malaria control activities [14]. Thirty-three districts have obtained support to implement educational programmes for medicine sellers and communities on the use of antimalarial medicines in the home from the Global Fund to fight AIDS, Tuberculosis and Malaria (GFATM). In April 2004, Kenya changed the first-line recommended antimalarial drug policy from Sulphadoxine/Sulphalene-pyrimethamine medicines (SP) to the artemesinin-based combination therapies (ACT), with a phased introduction through the formal government and mission sectors, to be followed by the private formal sector. The policy proposed ultimate inclusion of private medicine sellers, depending on early experiences with the change [15].

In this paper, we present data from household surveys of treatment seeking behaviour for recent childhood fevers and adult illnesses, and on malaria prevalence in well children under five years, in rural areas of three districts in Kenya. The data were collected during surveys conducted collaboratively by the Kenyan Medical Research Institute (KEMRI) Wellcome Trust Research Programme and the Division of Malaria Control (DOMC) in the Kenyan Ministry of Health (MOH) in 2002 and 2003 in districts with varying malaria transmission. The surveys aimed to inform the planning and evaluation of district-led training programmes for private medicine retailers and communities on malaria and its treatment. Data from the malaria parasite prevalence surveys are presented in this paper as markers for transmission pressure in the study sites, and for comparison with treatment-seeking behavior patterns. The findings are discussed with particular emphasis on adult OTC antimalarial use as an emerging challenge to programmes addressing this sector.

## Methods

### Study sites and population

**Figure 1:**
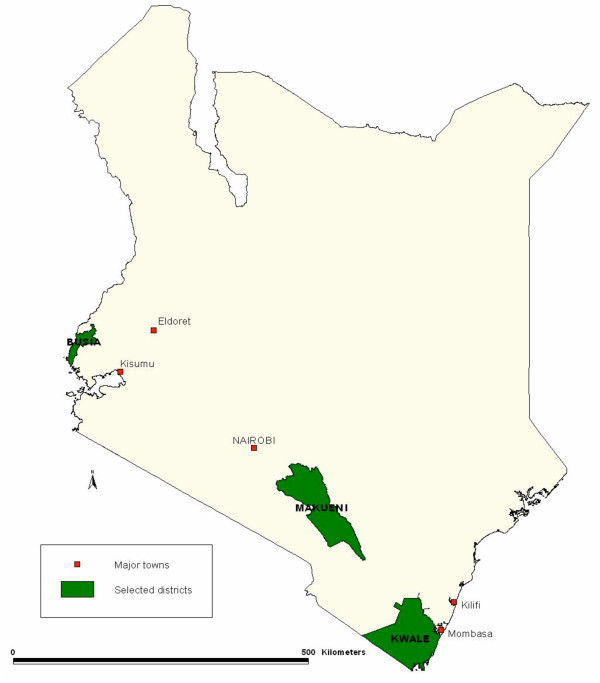
A map of Kenya showing the geographical locations of the three districts included in this study.

The Kenya DOMC selected three districts for study, Busia, Makueni and Kwale (Figure 1). Two districts (Kwale and Makueni) had already been identified as sentinel districts for the monitoring and evaluation of targets set in the KNMS, such as access to antimalarial drugs and coverage of ITNs [14]. Kwale (population 544,468) is a coastal district with high perennial malaria transmission, while Makueni (population 771,545) is a semi-arid area with seasonal transmission located midway between Nairobi and the coast. Busia (population 405,389) was chosen as a site of high perennial malaria transmission in Western Kenya. Within these districts, surveys were conducted in divisions identified by health management teams as potential sites for future retailer programmes. The divisions selected were all considered to have similar malaria incidence rates (from routine Health Information System data) and to contain typical remote rural areas. Priority was given to areas without existing governmental or non-governmental community based malaria control programmes. Ten study divisions were chosen in consultation with district health teams for random allocation to intervention (for early implementation) and control (for later implementation); two in Busia and four each in Kwale and Makueni. Table 1 describes the population and government health facilities within the study divisions.

**Table 1:** Population and number of health facilities in study divisions

Division	Population*	Number of government health centres	Number of government dispensaries	Number of private pharmacies
**Makueni district**				

Kathonzweni	65,738	2	5	2
Kalawa	26,333	1	2	2
Matiliku	38,867	2	2	4
Makindu	50,299	1	3	3

**Kwale district**				

Matuga	78,814	1	6	1
Kinango	78,433	0	7	2
Msambweni	230,648	3	20	4
Samburu	99,105	2	8	2

**Busia district**				

Matayos	60,365	1	2	3
Butula	104,450	2	1	11

*From National Census Data [46]

### Study design and method

This was a descriptive study based on cluster randomized cross sectional household surveys conducted during the peak malaria transmission season in each district. The methods used were surveys of reported treatment seeking behaviour for recent illness episodes in young children and adults, and malaria parasite prevalence surveys in a sub sample of well children using rapid diagnostic tests.

### Household surveys

In each study division, a list of National Census enumeration areas was used to construct a sampling frame and units for the household survey. Since some enumeration areas were much larger than others, to avoid weighting the chances of selection by population size, those with greater than 110 households were subdivided into smaller units prior to randomisation. In each district, a total of 4,000 households were identified in randomly selected enumeration areas (or subdivisions) of approximately 100 households. The sample size calculation was derived for planned future evaluations of the retailer training programmes, with a primary outcome of the proportion of childhood fevers treated with OTC antimalarials that included an adequate amount of the MOH recommended treatment. Since the future evaluations planned to include both intra and inter-district comparisons, sample sizes for the current household survey were calculated around comparisons of these proportions within districts, providing standard errors of 1.6% and 2.2% around estimations of 10% and 20% respectively (coefficient of variation 0.2) [16].

Locally recruited and trained field workers collected detailed information on treatment seeking behaviour for children under five years of age with a recent fever and for adults present in the household at the time of interview with a recent acute illness, using a structured questionnaire. Recent episodes were defined as those occurring within a two-week period of the interview. In this survey, two target age groups were identified; children under five who present the greatest vulnerability to severe disease, and individuals over the age of fourteen years, as an adult group likely to make an autonomous decision on treatment seeking. To counteract the potential for reporting bias, a pre-tested questionnaire developed and validated from previous studies was used [17], and field workers used drug charts containing samples of commonly used OTC medicines to aid recall and validate reports, and remaining packages or tablets were reviewed where possible. Data were checked for inaccuracies and inconsistencies daily before double entry and verification using FOXPRO version 6 (Microsoft Corp, Redmond, WA, USA). Adjustment was made for clustering at EA level using *Svy *data commands in STATA version 8 (Stata Corp, College Station, Texas, USA). Proportions for key outcomes are presented with 95% confidence intervals. Chi-square tests of association were used to compare proportions across the districts and analysis of variance (ANOVA) models were used to compare means. Since one of the most common OTC antimalarial medicines in use, amodiaquine (AQ), has a three-day regime of administration, episodes where treatment was started within three days of the interview were excluded.

### Malaria parasite prevalence surveys

Randomly identified sub-samples of 971, 963 and 980 children from the household survey in Busia, Kwale and Makueni districts, respectively, were invited to participate in a cross sectional survey of malaria parasite and anaemia prevalence. The prevalence of anaemia was assessed during the baseline as a marker for programme impact in the future, and data on haemoglobin concentrations are not presented in this paper. The sample size was based on a conservative estimate of 50% prevalence for moderately severe anaemia [18], with a confidence interval of 44% – 56%. Participants were screened at temporary local centers using rapid diagnostic tests for malaria (Uni-Gold ™ Trinity Biotech, Ireland). Children with parasitaemia and a history of fever and those with anaemia were provided with treatment in accordance with a treatment protocol approved by the national Scientific Coordinating Committee and the District Medical Officer of Health.

### Ethical approval

The Kenya National Scientific Steering and Ethical Committees and World Health Organization Ethics Review Committee granted approval for the study. The aim and purpose of all components of the study was discussed and agreed with local leaders. Verbal informed consent was sought for the household survey and information sheets including contact details were left in each home visited. Signed informed consent was obtained from parents or guardians for the malaria parasite prevalence survey. Reports on the findings of the surveys were sent to District Health Management Teams.

## Results

12,445 households were visited to interview 11,505 caregivers of children under five years and 19,914 adults across all the districts. Table 2 summarizes the number of households visited, interviews held, the prevalence of recent fevers in children or acute illnesses in adults, patterns of OTC medicines use in children and adults and parasite prevalence in each district. Parasite prevalence in Makueni was compatible with a hypoendemic pattern of malaria transmission, in contrast to the hyperendemic patterns seen in Kwale and Busia [19]. OTC medicines provided the commonest first treatment for fevers in children and acute illness episodes in adults. In general, children with fever treated through private retailers were more likely to be given an antipyretic than an antimalarial treatment, especially in Kwale and Makueni. Adults were more likely to buy antimalarials to treat their own illnesses than children were to receive these for febrile illnesses. The two most commonly used types of OTC antimalarials were the MOH first and second line recommended medicines at that time, SP and AQ, respectively, together accounting for 80.1% (95% CI 76.4, 83.3%) and 86.4% (95% CI 83.9, 83.3%) of all OTC antimalarials used in children and adults respectively. The remaining 19.9% of antimalarials used in children included chloroquine (16.6%), combinations of AQ and SP (2.1%) and others (1.3%). Among adults the remaining 13.6% of antimalarials included chloroquine (10.0%), combinations of AQ and SP (2.8%), and others (0.6%). The first line recommended medicine, SP, was more commonly used than AQ in one district for children (Busia, where 46.0% children using OTC AMs received SP) but in all other areas and age groups, AQ was either more likely or equally likely to be used. The adequacy of treatment was gauged in comparison to the national malaria guidelines on AM medicine use, shown in tables 3 and 4. Across all districts, single dose SP was used adequately by the majority (83.8%; 95% CI 78.7, 86.6) of adults and by nearly half (46.4%; 95% CI 39.5, 53.4) of children. Multiple dose AQ medicines were generally used inappropriately; 12.1% children (95% CI 16.3, 34.2) and 1.9% adults (95% CI 0.8, 4.2) took adequate amounts of AQ for their illness. The common pattern reported for AQ was single dose treatment.

**Table 2:** Number of households visited, prevalence of illnesses, use of OTC antimalarials and malaria parasite prevalence (%: 95% CI)

	Busia	Kwale	Makueni	All districts	P values *
Households visited	4017	4174	4254	12 445	-

**Children under 5 years**					

Number interviewed	3451	4081	3973	11 505	-
Recent fever	1437/3451 (41.7: 39.5, 43.9)	1770/4081 (43.3: 41.5, 45.2)	1216/3973 (30.8: 28.7, 33.0)	4423/11 505 (29.0: 28.1, 29.8)	<0.001
Fevers first treated with OTC medicines^†^	540/1437 (37.6: 35.1, 40.1)	898/1770 (50.7: 48.3, 53.1)	641/1216 (52.7: 49.8, 55.5)	2079/4423 (47.0: 45.5, 48.5)	<0.001
OTC users taking an AM^‡^	237/519 (45.7: 41.3, 50.1)	147/898 (16.3: 13.9, 18.9)	83/638 (13.3: 9.5, 17.9)	467/2057 (22.7: 20.9, 24.6)	<0.001
OTC SP users taking adequate dose^§^	68/141 (48.3: 37.9, 58.8)	19/39 (48.6: 32.5, 64.9)	11/31 (40.4: 19.0, 66.3)	98/211 (46.4: 39.5, 53.4)	0.415
OTC AQ users taking adequate dose	16/112 (14.2: 8.7, 22.2)	5/56 (9.1: 4.0, 19.5)	3/31 (10.7: 3.4, 28.9)	24/98 (12.1: 16.3, 34.2)	0.547
Rapid malaria test positive	805/971 (82.8: 79.0, 86.1)	695/963 (71.1: 64.4, 76.9)	34/980 (3.2: 2.1, 4.8)	1534/2914 (52.6: 50.8, 54.4)	<0.001

**Adults**					

Number Interviewed^∥^	6198	6750	6966	19 914	-
Recent illness**	1027/6198 (16.6: 14.4, 19.0)	1805/6750 (26.7: 24.9, 28.6)	1268/6966 (18.2: 17.1, 19.4)	4098/19 914 (20.6: 20.0, 21.1)	<0.001
First treated with OTC medicines	472/1027 (46.0: 42.8, 49.1)	1103/1805 (61.1: 58.8, 63.3)	753/1265^10^ (59.5: 56.7, 62.2)	2328/4097 (56.8: 55.2, 58.3)	<0.001
OTC users taking an AM	Not collected^††^	291/1081 (26.9: 21.3, 33.5)	384/745 (53.3: 48.6, 57.9)	675/1826 (36.9: 34.7, 39.2)	<0.001
OTC SP users taking adequate dose	80/102 (78.3: 68.4, 85.8)	75/95 (79.4: 68.3, 87.3)	157/179 (87.7: 81.5, 92.0)	312/376 (83.8: 78.7, 86.6)	0.07
OTC AQ users taking adequate dose	2/44 (4.4: 1.1, 16.6)	0/148	5/174 (2.4: 0.9, 6.0)	7/336 (1.9: 0.8, 4.2)	0.07

*. Chi-square test of association for differences between districts

†. Includes episodes where the individual was visited 3 days or more after treatment begun to exclude part courses of amodioquine (also see methods); OTC medicines includes those bought from general shops, chemists or mobile vendors and those kept at home from. 2. All adults available in the home at the time of visit

‡ There were 21 episodes in Busia and 3 in Makueni where OTC medicine could not be identified

§. Adequate dosage is according to MOH recommendations (see table 3 and 4): over dosage occurs when more and under dosage when less than the recommended amount of the drug is given

∥ All adults available in the home at the time of visit

**. In Busia, the frequency refers to the number of adults reported "perceived malaria" whereas in Kwale and Makueni this represents the number of adults with a recent acute illness of any type, excluding trauma.

†† Data on the proportion of acute illnesses in adults where an AM was used was not collected in Busia.

**Table 3:** National guidelines on dosage by age for SP medicines. [1 tab = 500 mg sulphadoxine, 25 mg pyrimethamine; 5 ml = 250 mg sulphadoxine, 12.5 mg pyrimethamine]

				Total no spoons syrup	
				
Age	Days given over	Total no tabs	Total in mg*	tsp (5 ml)	tbs (10 ml)
<11 months	1	1/2 – 3/4	250 – 375	1 – 1 1/2	1/2 – 3/4
12 – 59 months	1	1 – 1 1/4	500 – 625	2 – 2 1/2	1 – 1 1/4
5–8 years	1	1 1/2 – 1 3/4	750–875		
9–14 years	1	2–2 3/4	1000–1375		
15+ years	1	3	1500	-	-

*for sulpha component

**Table 4:** National guidelines on dosage by age for AQ medicines. [1 tab = 200 mg AQ base; 5 ml = 50 mg AQ base]

				Total no spoons syrup	
				
Age	Days given over	Total no tabs	Total in mg	tsp (5 ml)	tbs (10 ml)
< 6 months	3	3/4 – 1	150 – 200	3 – 4 1/2	1 1/2 – 2
6 – 11 months	3	1 1/4 – 1 3/4	250 – 350	5 – 7 1/2	2 1/2 – 3 1/2
12 – 47 months	3	2 – 2 1/4	400 – 450	7 1/2 – 9 1/2	4 – 5
48 – 71 months	3	2 1/2 – 3 1/2	500 – 700	10 – 14 1/2	5 – 7
15 – 16 years	3	4–6 1/4	800–1250	-	-
16 + years	3	6 1/2–7 1/2 -	1300–1500	-	-

Table 5 presents data on the second actions taken by those who used OTC medicines as a first step. For this analysis, users who purchased medicines more than once from a shop before moving to a second resort to treatment were grouped as single action. 82.9% children and 88.9% adults using OTC medicines as a first measure took no further action. Amongst the group taking no further action, 9.7% children (133/1377) and 13.5% adults (209/1547) reported remaining unwell, while the remainder reported full or almost complete recovery (where the latter was defined as having no intention to seek further treatment). For the minority that took further action, most subsequently visited a formal provider but this action was generally not taken within 48 hours of onset of illness. However, by 72 hours, 66.9% (95% CI 61.1, 72.3) of children in this group of self-referrals reported consultation at a government or private clinic.

**Table 5:** Actions following first use of OTC medicines (%: 95% CI)

	Busia	Kwale	Makueni	Overall	P
**Children**					

No further action after OTC	511/540 (94.6) (92.3, 96.6)	657/837 (77.9) (73.3, 82.0)	469/598 (78.3) (73.4, 82.6)	1637/1975 (82.9) (81.1, 84.5)	<0.001
2^nd ^action is use of clinic	24/540 (4.4) (2.8, 6.5)	153/837 (18.3) (15.7, 21.1)	107/598 (17.9) (14.9, 21.2)	284/1975 (14.4) (12.8, 16.0)	<0.001
Visits clinic within 48 h*	8/24 (33.3) (15.6, 55.3)	37/153 (24.2) (17.6, 31.7)	41/107 (38.3) (29.1, 48.2)	86/284 (30.3) (24.9, 35.9)	0.05

**Adults**					

No further action after OTC	: 427/453 (94.3) (91.7, 96.2)	923/1065 (86.4) (83.1, 89.0)	637/717 (88.6) (85.6, 91.0)	1987/2235 (88.9) (87.5, 90.1)	<0.001
2^nd ^action is use of clinic	: 4/453 (0.9) (0.2, 2.2)	104/1065 (9.8) (8.0, 11.7)	63/717 (8.8) (6.8, 11.1)	171/2235 (7.7) (6.5, 8.8)	<0.001
Visits clinic within 48 h	3/4 (75.0) (19.4,99.3)	32/104 (30.1) (22.1, 40.6)	11/63 (17.5) (9.0, 29.1)	46/171 (26.9) (20.4, 34.2	0.015

*. Within 48 hours of the onset of the illness

## Discussion

Given the high global burden of young childhood disease and deaths attributed to malaria [1,2], early effective treatment of childhood fevers has become a main focus within global and national malaria control strategies [3,13,14]. Such strategies highlight the role of private medicine retailers to promote early effective home treatment of childhood malaria in endemic settings [13]. More recently, the introduction of ACTs as first line treatment for malaria in many countries in sub Saharan Africa has led to debate on the role of community-based mechanisms for delivery of malaria treatments [20-23]. Understanding patterns of OTC medicine use is important to assess the opportunities and challenges of programmes for private medicine retailers, but there is limited detailed data on adult OTC antimalarial use in the literature [23-26]. This study reports on patterns of OTC medicines use from large-scale surveys including child and adult populations across three districts in Kenya with varying malaria endemicity.

In interpreting these results, it is important to consider limitations of the study methods. The data on treatment seeking behaviour were collected from reports concerning recent illness episodes. While standard practices were used to ensure that these were as accurate as possible (careful training and supervision of interviewers, internal validity checks in questionnaires, use of locally constructed drug charts with packaging and logo visuals to aid recognition, and inspection of left-over tablets), this type of data is always open to recall and reporting biases. In the household survey, for pragmatic reasons, histories were elicited for all children within a household who met inclusion criteria for age, including siblings. Theoretically, this may lead to clustering of data within households for which no allowance has been made in the analysis. Confidence intervals could therefore be wider than those stated here, although these are generally small given the size of these surveys. There is some evidence that intra-household clustering would have a negligible effect; a study in rural Tanzania found no difference in the outcome of an analysis of treatment seeking behaviour for all young children and for only one randomly selected child within that household [27]. Finally, these data were collected from surveys conducted in 2002 and 2003, at a time when the recommended first line antimalarial medicine in Kenya was SP. Practices of drug use may have changed over time, and care should be taken in extrapolating these results to understand use of other antimalarial medicines. However, the pattern of poor adherence with multidose medicines is not a new or isolated phenomenon [28,29] and many factors underlying medicine use for acute illnesses (cost, complexity of regimen and rate of response) are likely to be generalisable.

The data presented here concur with earlier descriptions of the popularity of private medicine sellers as an early treatment option for childhood fevers, and the inappropriate ways in which OTC antimalarial medicines are generally used in young children [17,23,30-33]. The study further illustrates that adults with a recent acute illness were more likely to use an antimalarial medicine than were young children to receive them for a febrile illness, even in hypoendemic districts. These findings reinforce the need to intervene in a sector that is chaotic but potentially offers an opportunity to improve early fever treatment practices in children. They also point to shortcomings in applying current global home malaria management strategies that focus primarily on young children to private retail sector interventions. While the under five age group experiences the greatest burden of malaria disease in endemic settings, adult use of OTC antimalarials may represent a greater challenge to strategies to optimise their use [34].

There is limited information on adult use of OTC antimalarial medicines. Several studies have addressed the frequency of OTC antimalarial use in adult populations in specific settings, but there is less information on the way that these medicines are used, or from large-scale surveys in areas of varying malaria endemicity. A study of OTC antimalarial use in a highland area of low seasonal malaria transmission in Kenya described a similar excess of adult over paediatric use (24% of adult and 14% of child OTC users purchased an antimalarial) [24]. High use of OTC antimalarials has been described for older school children in Kenya [26], as have similarities between adult and child use of OTC antipyretic and antimalarial medicines in a qualitative study in Ghana [25].

Adult over-use of antimalarials is an important phenomenon because of the potential for wastage of resources where adults have a lower risk of malaria than children. The extent of over-use is highlighted by the popularity of these medicines even in areas with low and seasonal malaria transmission. Adult over-use affects estimations of the volume of malaria medicines used in the private retail sector and therefore has an economic implication for governments and households, particularly in anticipation of, or following, the introduction of ACTs [24]. There may be an impact of under dosing on the development of drug resistance. Although the relationship between these is not straightforward, resistance is likely to be selected for where partially effective treatments result in recrudescence of infection [35]. If under dosing contributes to increased resistance of malaria parasites to antimalarial medicines, the widespread existence of such practices amongst adults as well as children can only increase this effect.

For both adults and children, there are clear differences in adherence to SP and AQ medicines, with adherence to SP being much higher than AQ. This finding is likely to be a result of the different regimes for administration, with SP medicines being given in a single dose in contrast to the 3-day AQ regimen. There is evidence that other multiple dose medicines, such as chloroquine, have commonly been used as single doses in the past, including in Kenya [36]. Adherence to multidose ACTs may present a similar problem, particularly given the speed of response of symptoms, such as fever, to this group of medicines. Although high adherence to ACTs has been documented in the formal sector [37,38], under-dosing is often a cost-saving strategy [32,39] and is, therefore, particularly likely to affect private retail sector treatments where the costs are directly related to the quantity of medicine obtained. This incentive to cut down on full courses of medicines may plausibly be greater for adults than for young children in keeping with the larger amounts of medication that they require. In this survey, adult AQ adherence is considerably worse than that reported for children.

A further pattern of interest emerging from the study concerns movement between private medicine sellers and trained health workers. Amongst the minority (14.4%), who sought further treatment after using OTC medicines, most carers (approximately 84%) chose to visit a trained health worker. Self referral to such a facility did not generally occur within 24 hours of onset of illness, as recommended by the Roll Back Malaria initiative [13], but the majority did seek further assistance by 72 hours. Kemble et al studied this pattern of self referral in an urban population in Kampala, finding a much higher proportion (42%) of child OTC users were subsequently taken to a formal provider, although with similar timing [23]. In an epidemic area in Uganda, health facilities were reported as the commonest first line treatment for both adults and children with suspected malaria [40]. More research on these patterns, and the factors underlying self-referral behaviour would help to elucidate opportunities for optimising these practices.

Programmes for private medicine retailers in malaria control have been based on pragmatic principles, and these still obtain. The challenges of working with informal private providers have been well articulated at the policy level, including the size of the sector in many countries, lack of regulatory mechanisms and capacity, lack of organizational structures, multiplicity of brands, inadequate quality of OTC medicines, difficulties in diagnosing malaria clinically and commercial interests of retailers [5,12,20,41,42]. However, unit dose pre-packaging interventions have already been shown to improve adherence with multidose medicines, and may prove effective for antimalarial medicines in the private retail sector (10, 41). The opportunities provided by this sector have also proved compelling, including sustainability, cost and coverage [31,36,43,44], particularly given the shortcomings reported in the public sector, reviewed recently by Zurovac and Rowe [45]. Mills et al illustrate this pragmatism writing that "...training and investment in formal sector providers (public and private) and restructuring of the market so as to strengthen the purchasing and regulation functions of government, may displace the informal sector, but this is likely to be a very long term process" [12]. The data presented in this paper show that the group of mothers who visit a health facility as a second option is a minority, but that this self-referral occurs relatively quickly, even in rural settings. Kemble suggests that faster movement to a health centre may be achievable with the provision of adequate, acceptable and affordable services and a better public understanding of the need for rapid treatment of childhood fevers [23]. This data also indicate that interventions directed at increasing usage of health facilities may be feasible and play an important part in improving early effective malaria treatment. With recent increased investment in malaria control activities, further research is needed to establish the extent to which the introduction of free, effective antimalarial treatment in government health facilities, and the wider availability of free or affordable ITNs can influence the balance of choice for users between trained public and untrained commercial providers in the future, taking account of socio-economic and geographic differences. Where OTC antimalarial use remains a popular option, greater targeting of antimalarial medicines to high risk populations, and away from the current predominantly adult market, will be an important challenge for private medicine retailer programmes in the future [12,23,24].

## Conclusion

The findings of this survey across three districts of differing malaria endemicity in Kenya confirm the common use of OTC medicines for fever in children and acute illnesses in adults, as well as the widespread inappropriate use of these medicines. They point to the frequent use of OTC antimalarial medicines in adults, irrespective of disease transmission rates, and the high risk that this will be accompanied by under dosing. A minority of OTC users subsequently seek further treatment through the formal sector, but many of these self-referrals occur within 72 hours of onset of illness even in rural situations. More research is needed to assess the impact on the popularity of private medicine sellers of free malaria treatment in public health facilities for children under five years and increased community coverage with treated bed nets. Improved targeting of OTC antimalarial medicines to high risk groups, better communication strategies on adult as well as children's dosages, and facilitating appropriate referral to trained health workers are important challenges to private medicine seller programmes.

## Authors' contributions

Timothy Abuya was involved in the design of the research, was primary supervisor of the survey work, analysed the data and prepared the first draft of the manuscript. Wilfred Mutemi was involved in the design of the research, supported the survey work and contributed to the writing of the manuscript. Karisa Baya was involved in the design of the research, was a major contributor to the survey work and contributed to the writing of the manuscript. Sam Ochola was involved in the design of the research, supported the survey work and contributed to the writing of the manuscript. Greg Fegan was involved in the design of the research, supervised the analysis of data and contributed to the writing of the manuscript. Vicki Marsh was responsible for the design of the research, planning and supervising survey work and contributed to the writing of the manuscript.
